# Preventing polio from becoming a reemerging disease.

**DOI:** 10.3201/eid0707.017728

**Published:** 2001

**Authors:** W R Dowdle, S L Cochi, S Oberste, R Sutter

**Affiliations:** Task Force for Child Survival and Development, Decatur, Georgia, USA. wdowdle@taskforce.org


### Preventing Polio from Becoming a Reemerging Disease

Walter R. Dowdle,* Stephen L. Cochi,† Steve Oberste,† and Roland Sutter†
*Task Force for Child Survival and Development, Decatur, Georgia, USA, and †Centers for Disease Control and Prevention, Atlanta, Georgia, USA

The global effort to eradicate polio has become the largest public health initiative in history and is spearheaded by the World Health Organization, Rotary International, the Centers for Disease Control and Prevention, and UNICEF (United Nations Children's Fund). During 1999, extraordinary progress continued, with the number of polio-endemic countries declining to 30 from 50 in 1998. Of the three poliovirus types, poliovirus type 2 has reached the verge of extinction, with the only known remaining foci existing in northern India. Polio incidence declined to the lowest levels ever in 1999, although the number of reported cases (7,012) increased slightly due to improvements in surveillance and polio outbreaks in Angola and Iraq. Existing challenges in the initiative include maintaining effective activities, gaining access to children in conflict-affected countries, and sustaining political and financial support until certification is achieved in 2005. Maintaining sufficient supplies of oral polio vaccine emerged as an additional challenge during 1999, resulting from marked acceleration of immunization activities. The public-private sector partnership supporting the initiative expanded in 1999 to include the Bill and Melinda Gates Foundation, Ted Turner's United Nations Foundation, the World Bank, Aventis Pasteur, and De Beers.

### Cessation of Polio Vaccination

Following certification of polio eradication by the year 2005 or shortly thereafter, the public health community and policy makers will be faced with the decision of how and when to stop polio vaccination. The benefits of ceasing vaccination are well defined (i.e., annual savings of U.S. $1.5 billion in direct global vaccination costs; the possibility of directing these savings to other health priorities; and eradication of vaccine-associated paralytic poliomyelitis cases). The risks are obvious. If poliovirus is reintroduced into a susceptible population, a catastrophic epidemic of paralytic disease, disability, and death could ensue. Poliovirus could reemerge through 1) reintroduction of poliovirus from a laboratory; 2) prolonged replication in immunodeficient patients; and 3) persistent transmission of vaccine-derived virus in populations. The probability of vaccine-derived poliovirus remaining in circulation is impossible to estimate directly. However, the basic reproductive number or BRN (derived from proportion excreting virus, duration of excretion, virus titer in stool) for vaccine-derived poliovirus is lower than for wild-type polioviruses (typically between 2 and 5 in industrialized countries and 10 and 15 under conditions of poor hygiene in tropical countries). Additional data exist from outbreak investigations, molecular sequencing of polioviruses, and studies of poliovirus persistence following mass vaccination campaigns (e.g., Hungary, Finland, and Cuba). Although the currently available data are encouraging (i.e., decreasing BRN and 3 to 6 months' duration of circulation), substantial gaps in knowledge still exist, including the probability of continued virus circulation in populations with poor hygiene. These gaps need to be addressed to ensure that the best available scientific data will be available for decision-making.

### Laboratory Containment of Wild Polioviruses

Global documentation of laboratory containment of materials infected or potentially infected with wild poliovirus is a key component in the decision to stop vaccination. The last smallpox case occurred not in Somalia in 1977, but in England in 1978. The virus was transmitted through a faulty ventilation system from a laboratory to a nearby office, where it infected a person. Like smallpox virus, the only remaining sources of wild virus will be in the laboratories once the virus has been eradicated from the natural environment. The reported transmission of wild poliovirus from a vaccine production facility, presumably through an infected worker, to the community underscores the need for increased containment once wild poliovirus has been eradicated (1).

Infectious or potentially infectious poliovirus materials may be present in a wide range of laboratories, including clinical diagnostic, environmental, research, and teaching. The types of materials that might contain wild poliovirus include clinical (e.g., diagnostic specimens or unidentified enteroviruslike isolates), research (e.g., wild poliovirus strains or derivatives, full-length poliovirus RNA, or cDNA containing full capsid sequences), and environmental (sewage). Because of its high rate of subclinical infections, poliovirus may be found in fecal specimens collected for other purposes. For example, fecal or sewage samples collected in polio-endemic countries for nutritional and environmental studies or studies of other viral, bacterial, or parasitic diseases may contain wild poliovirus.

The *WHO Global Action Plan for Laboratory Containment of Wild Polioviruses* (2) was published in 1999. The plan is linked to the eradication progress. In the preeradication phase (the present), laboratories are required to implement safe handling procedures for materials infected or potentially infected with poliovirus (biosafety level [BSL] 2/polio). Countries must establish national inventories of laboratories holding such materials. Completion of preeradication activities is required before a region can be certified polio-free.

Address for correspondence: Walter R. Dowdle, 750 Commerce Drive, Suite 400, Decatur, GA 30030 USA; fax: 404-371-1087; e-mail: wdowdle@taskforce.org

The posteradication phase (high containment) begins 1 year after identification of the world's last case. At that time, laboratories holding wild poliovirus stocks and potentially infectious materials must either place all materials under appropriate biosafety conditions, transfer important virus isolates to WHO interim repositories, or render all wild poliovirus materials noninfectious. Documentation of containment compliance by all regions is required for global certification of poliovirus eradication. For countries that intend to stop all poliovirus vaccination, work with materials that could cause infection with wild poliovirus must be conducted under BSL 4 containment. High containment (BSL 3/ polio) will be required for work with vaccine-derived viruses.

High-level political involvement and multi-sector commitments, including departments of health, defense, education, environment, and private industry are essential to achieving and maintaining global containment of wild poliovirus.

### References

1. Mulders MN, Reimerink JHJ, Koopmans MPG, van Loon AM, van der Avoort HGAM. Genetic analysis of wild type poliovirus importation into The Netherlands (1979-1995). J Infect Dis 1997;176:617-24.
2. World Health Organization. Global action plan for laboratory containment of wild polioviruses. Geneva, Switzerland: World Health Organization; 1999.

### GIDEON: A Computer Program for Diagnosis, Simulation, and Informatics in the Fields of Geographic Medicine and Emerging Diseases

**Stephen A. Berger**
Tel Aviv Medical Center, Tel Aviv, Israel

Over 300 infectious diseases occur and are challenged by over 250 drugs and vaccines. Fifteen hundred species of pathogenic bacteria, viruses, parasites, and fungi have been described, and printed media can no longer keep up with the dynamics of diseases, outbreaks, and epidemics in "real time." Although electronic media have given us unlimited information access, the search for meaningful data is confusing and time-consuming. Global Infectious Diseases and Epidemiology Network (GIDEON) is a computer software program that was developed for disease simulation and informatics in the fields of geographic and travel medicine.

GIDEON is currently used in 1,500 sites in 45 countries: health ministries, military installations, travel clinics, libraries and student teaching modules, clinical departments, laboratories, and missionary agencies. The program consists of four components. The first generates a Bayesian ranked differential diagnosis based on signs, symptoms, laboratory tests, country of origin, and incubation period and can be used for diagnostic support and simulation of all infectious diseases in all countries. In a blind trial conducted on 495 patients, the correct diagnosis was included in the differential diagnosis list in 94.7% of cases (sensitivity) and displayed as the first disease in the list in 75% (specificity).

The second component presents the epidemiology of individual diseases, including their global effects and status in each of 205 countries and regions. All past and current outbreaks are described in detail, and a web-based version under development will allow for daily updating online. The user may also access a list of diseases related to any agent, vector, vehicle, reservoir or country or any combination of all five (i.e., a list of all mosquito-borne viruses of Brazil which have an avian reservoir).

The third module is an interactive encyclopedia which includes information on the pharmacology, use, testing standards, and global trade names of all antiinfective drugs and vaccines.

The fourth module is designed to identify all species of bacteria, mycobacteria, and yeasts. The database includes 50 to 100 additional taxa that may not appear in standard texts and laboratory databases for several months. Other options allow the user to add data relevant to his own institution, electronic patient charts, material from the Internet, important telephone numbers, drug prices, antimicrobial resistance patterns, and other information. This form of custom data input is particularly useful when running GIDEON on institutional networks because software administrators can use it to disseminate and file information relevant to their own institution for use by all computers on their network. The data in GIDEON are derived from all peer-reviewed journals in the fields of infectious diseases, pediatrics, internal medicine, tropical medicine, travel medicine, antimicrobial pharmacology, and clinical microbiology; a monthly electronic literature search based on all relevant terms in GIDEON (e.g., diseases, drugs, etc.) all available health ministry reports (both printed and electronic); standard texts; and abstracts of major meetings. Further details regarding the program are available at http://www.cyinfo.com.

Address for correspondence: Stephen A. Berger, 6 Weitzman Street, Tel Aviv 64239, Israel; fax: 972-3-6132892; e-mail: mberger@post.tau.ac.il
